# Insights into pelvic insufficiency fracture following pelvic radiotherapy for cervical cancer: a comparative review

**DOI:** 10.1186/s12905-024-03099-8

**Published:** 2024-05-23

**Authors:** Munima Haque, Md Sakib Hossen

**Affiliations:** 1 Biotechnology Program, Department of Mathematics and Natural Sciences (MNS), School of Data and Sciences (SDS), BRAC University, Kha-224, Merul Badda, Dhaka, 1212 Bangladesh; 2 Department of Biochemistry and Molecular Biology, Primeasia University, Banani, Dhaka, 1213 Bangladesh

**Keywords:** Pelvic insufficiency fractures, Cervical cancer, Radiotherapy, Risk factors, Treatment, Pathobiology

## Abstract

**Background:**

Radiotherapy (RT)-induced pelvic insufficiency fractures (PIF) are prevalent in patients with cervical cancer. Inconclusive studies on PIF after cervical irradiation create uncertainty. This review examined PIF after RT in cervical patients, including its pathobiology, likely locations of fractures, incidence, clinical symptoms, and predisposing factors. We further discussed study limitations and therapeutic possibilities of PIF.

**Methods:**

The following online resources were searched for relevant articles: Google Scholar and PubMed. The keywords ‘pelvic insufficiency fractures’, ‘cervical carcinoma’ and ‘cervical cancer’, as well as ‘chemoradiotherapy’, ‘chemoradiation’, and ‘radiotherapy’, were some of the terms that were used during the search.

**Results:**

Patients with PIF report pelvic pain after radiation treatment for cervical cancer; the incidence of PIF ranges from 1.7 to 45.2%. Evidence also supports that among all patients treated with pelvic radiation, those who experienced pelvic insufficiency fractures invariably had at least one sacral fracture, making it the most frequently fractured bone in the body. Menopausal status, weight, BMI, age, and treatments and diagnosis modalities can influence PIF during radiotherapy.

**Conclusions:**

In conclusion, our comparative review of the literature highlights significant heterogeneity in various aspects of PIF following radiation for patients with cervical cancer. This diversity encompasses prevalence rates, associated risk factors, symptoms, severity, diagnosis methods, preventive interventions, and follow-up periods. Such diversity underscores the complexity of PIF in this population and emphasizes the critical need for further research to elucidate optimal management strategies and improve patient outcomes.

## Introduction

Cervical cancer, the fourth most common cancer among women worldwide and the seventh most common cancer overall, poses a significant public health challenge due to its high incidence and mortality rates. In 2022, it was expected that 660 000 women around the world were diagnosed with cervical cancer, and around 350 000 women passed away as a direct result of the circumstances [1]. Diagnostic imaging techniques, including computed tomography (CT), magnetic resonance imaging (MRI), chest X-rays, and positron emission tomography (PET), are used to determine the stage of the disease and provide direction for treatment decisions [2, 3]. There are various treatment options available for cervical cancer, including surgery, radiation therapy, chemotherapy, and targeted therapy. The choice of treatment is based on the specific patient’s circumstances [4]. Radiation therapy, often used alongside surgical techniques to treat cervical cancer, has been linked to an increased risk of pelvic insufficiency fractures (PIF). Studies have shown that women with pelvic malignancies who undergo irradiation have a higher risk of PIF compared to those who do not undergo irradiation [3, 5–13].

Pathological insufficiency fractures occur when bone undergoes failure under normal physiological loads, mainly affecting weight-bearing regions such as the pelvis. Contributing factors to PIF include osteoporosis, vitamin D deficiency, age, rheumatoid arthritis, and extended use of corticosteroids or bisphosphonates [7, 14–19]. The occurrence of PIF (post-irradiation fractures) after radiation therapy is uncertain; however, symptomatic fractures often appear within one year after starting treatment [4, 19]. However, there is a lack of definitive information regarding the occurrence of PIF and the factors that contribute to it. This review aims to provide a comprehensive overview of the existing literature on PIF after radiation therapy for cervical cancer. It will cover various aspects, such as the underlying causes, locations of fractures, rates of occurrence, symptoms, factors that increase susceptibility, available treatment options, and limitations of current research [5, 20–24].

## Methodology

**Fig. 1:**
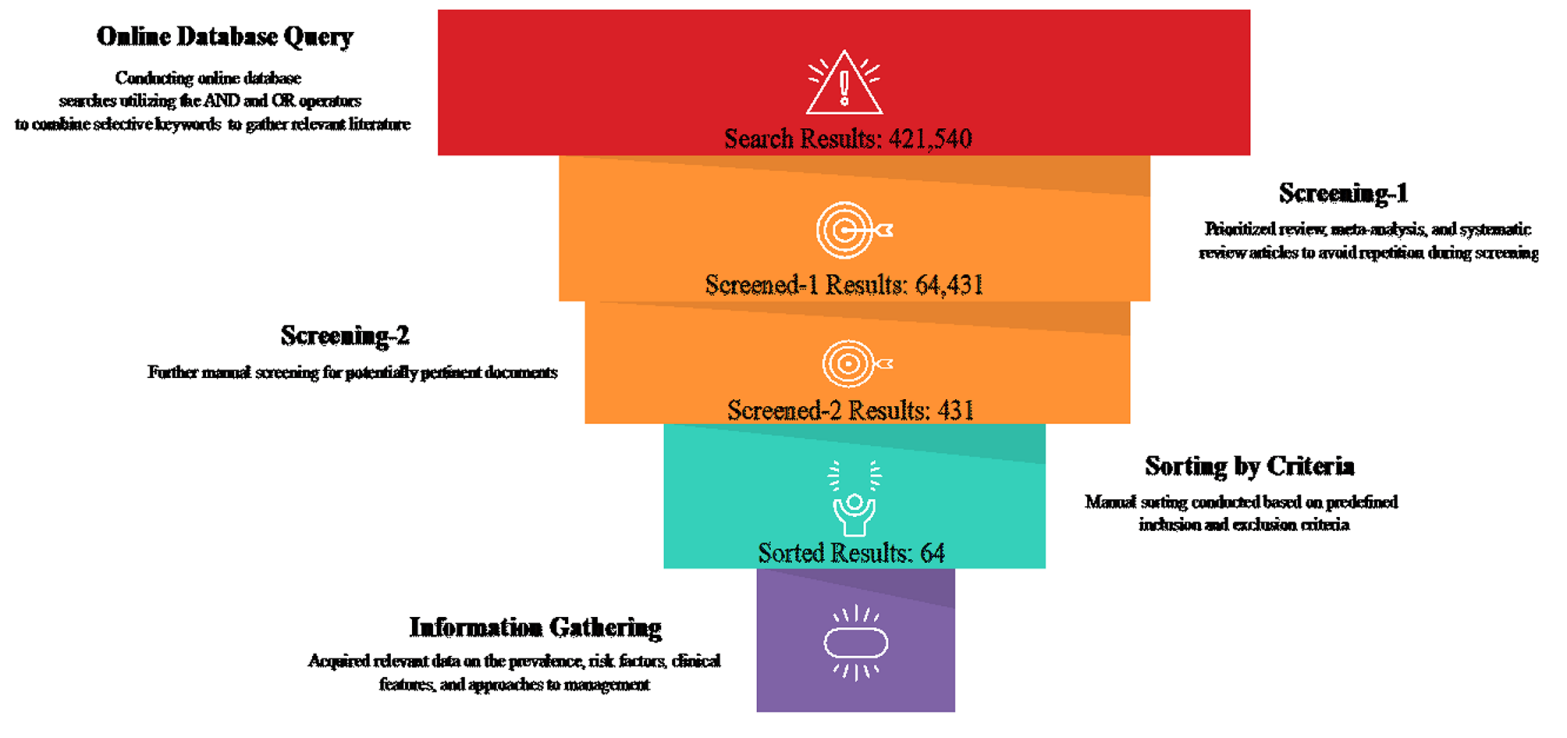
Approaches to browsing the literature and identifying pertinent research

The approaches used to explore the literature and find relevant studies are summarized in Fig. 1. In more detail, the following online resources were searched for relevant articles: Google Scholar and PubMed. The keywords ‘pelvic insufficiency fractures’, ‘cervical carcinoma’ and ‘cervical cancer’, as well as ‘chemoradiotherapy’, ‘chemoradiation’, ‘radiotherapy’, and ‘postoperative’ or ‘post-operative’, were some of the terms used during the search. To gather more literature, we searched the reference lists of previously published reviews and studies that were included in the review. In addition, a manual search approach was carried out as part of this investigation in order to identify other relevant citations that were published in articles. We did not limit our search to any particular time period to collect as much information as possible about PIF after pelvic radiotherapy for cervical cancer.

For this review, we considered studies that met one or more of the following conditions: (1) treated pathologically confirmed cervical neoplasms; (2) documented the incidence, clinical characteristics, and risk factors of PIF after RT; (3) presented a clear overview of the distribution of PIF, (4) articulated treatments to manage PIF complications; and (5) highlighted the limitations of the study.

Studies were not included if (1) they reported only secondary data (such as reviews, study procedures, remarks, or communications); (2) essential data could not be extracted; or (3) they were not published in English.

Our study has one limitation, that we did not take into account how the diagnosis and treatment process was set up. This is due to the fact that different techniques for identifying PIF employ different methodologies and degrees of sensitivity. Only the existence of PIF was verified; the approach itself was not explored.

## Discussion

### Theorized mechanism of PIF after radiation

Radiation either directly damages bone tissues or indirectly affects vascular alterations [25]. Because of this, PIF can later manifest itself as a consequence of pelvic radiation therapy administered to people with cervical cancer. In a direct pathway, the three major cells that make up bone, osteoblasts, osteocytes, and osteoclasts, can be impaired by radiotherapy [26]. The bone matrix, whose elements are crucial for providing bones with strength, is produced by osteoblasts. In light of this, radiation has the potential to produce osteopenia by reducing collagen formation and alkaline phosphatase activity, making it difficult for bones to cope with the demands of daily life [20, 25, 27].

**Fig. 2:**
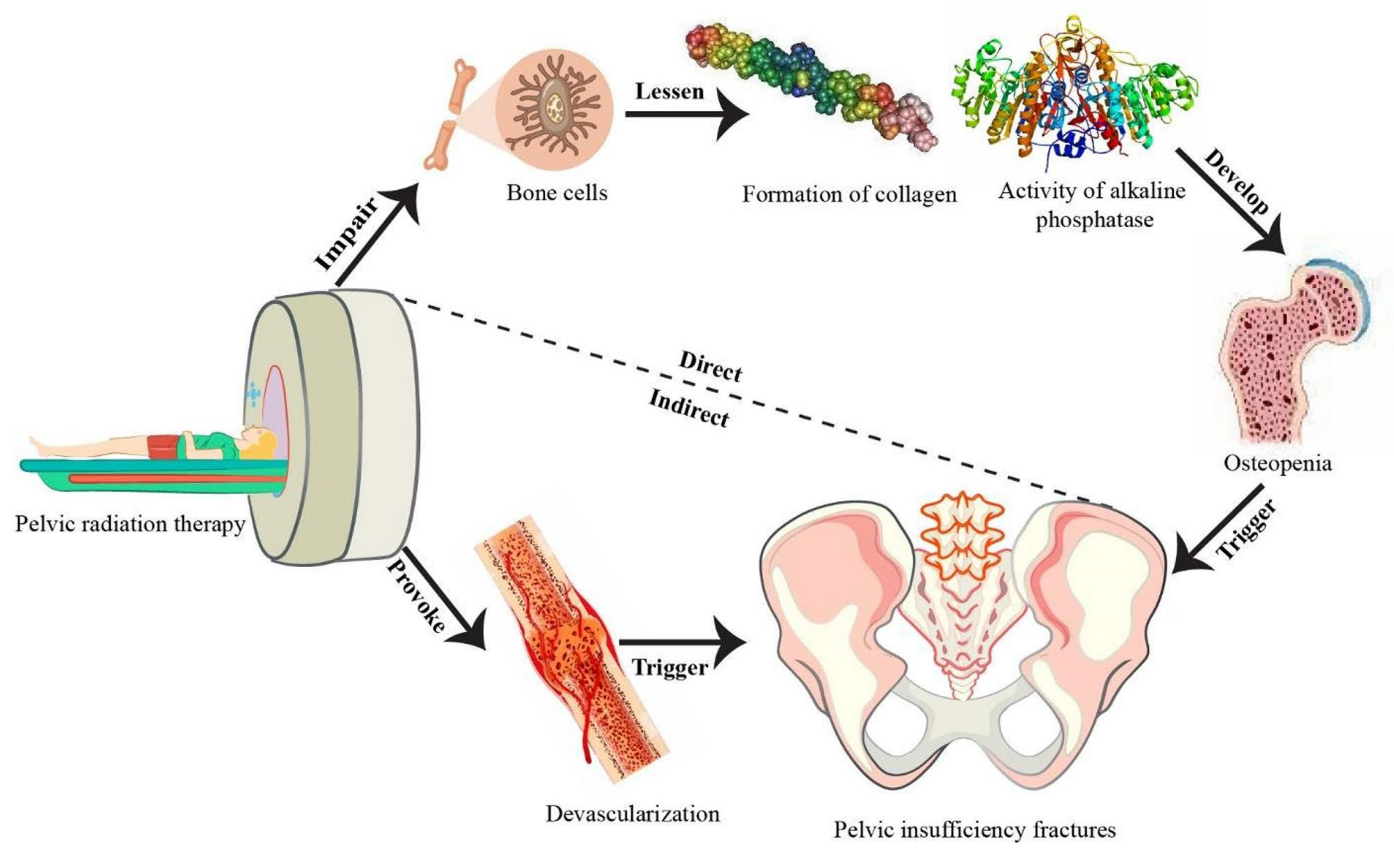
A conceptual diagram illustrating how RT promotes PIF development in cervical cancer patients

The theorized mechanism for the indirect pathway postulated that irradiation has an effect on local circulation, which therefore inhibits bone remodeling and turnover [5, 28]. Furthermore, radiation-induced devascularization of the bone increases the risk of fracture by denying vital nutrients to bone cells in the blood and causing additional bone loss [26, 29]. Figure 2 shows a schematic depiction of the mechanisms by which RT gradually promotes the growth of PIF in patients with cervical cancer.

### Observation of the literature on potential fracture locations

There is evidence to suggest that sacral fractures occur simultaneously with pubic fractures. These data point to the sacrum as the site of the initial mechanical breakdown, followed by the pubic fracture [30]. The consistency of the pelvic fracture site is one of their most distinguishing characterisctics. Sacral alae have been fractured in a vertically, parallel to the sacroiliac joints. They sit on the side of the lumbar spine, close to its edges. This pattern of stress concentrations indicates that the weight of the body, as conveyed by the spine, may have contributed to the fracture. Cooper et al. conducted a study on the subject, and found that two of the displaced fracture patients in the study had both normal vertical fractures and a transverse component. This research provides more evidence that the transverse fracture develops as a secondary result of stress on the already broken sacrum [31].

Altogether, the sacrum was the bone most frequently implicated bone for fractures [32]. When it comes to specific areas, the sacroiliac joints (SI) are the most common targets of PIF [22, 33–36]. These results demonstrated that successive pelvic bone fractures are the result of the initial mechanical failure [22, 33]. Likewise, Ramlov et al. determined that the sacrum was the most common site of fracture (77%) and that all patients had at least one sacral fracture. In 74% of all cases, the sacroiliac joint was shown to be the cause of the fracture in the sacrum [19]. Alternatively, Kwon et al. observed that in their study, sixty-one patients (61%) experienced numerous PIFs, and among them, forty (40%) had bilateral symmetric lesions of the sacral alae. Up to 85 patients (85%) were affected in the sacrum [22].

**Fig. 3:**
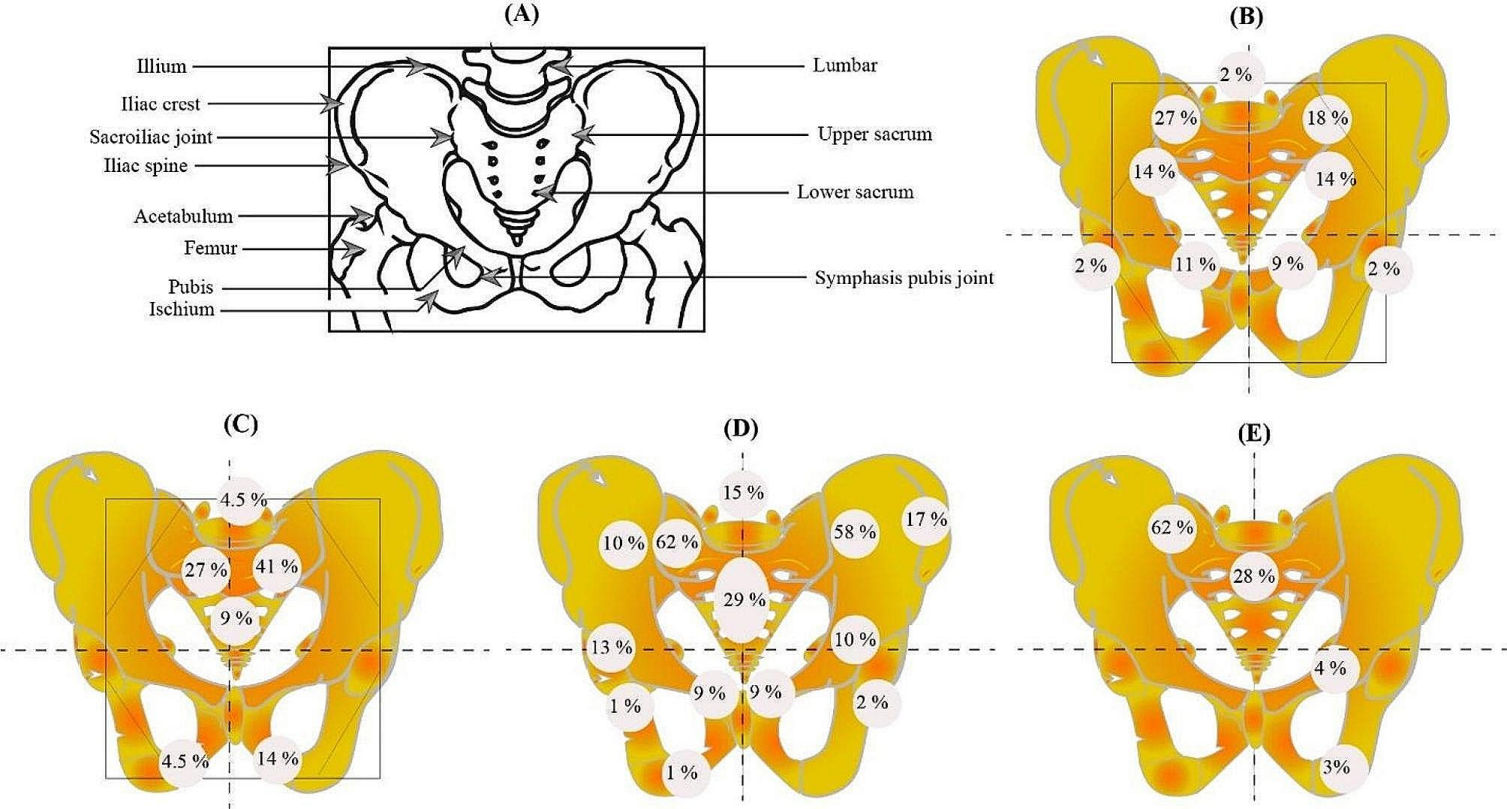
Ventral view of the pelvic region and the schematic distribution of PIF after RT, according to previous studies. (**A**) Diagrammatic representation of the female pelvic girdle. (**B**) PIF was observed at the sacral-iliac joints (32 sites, 72%), pubis (9 sites, 20%), acetabula (2 sites, 4%), and lumbar spine (1 site, 2%), according to Tokumaru et al. (**C**) The sacroiliac joint, which was shown in 15/22 fractures (68%), and pubic bone, which was seen in 4/22 (18.5%), were the most common fracture sites, according to Shih et al. (**D**) Sixty-one patients (61%) who experienced multiple pelvic insufficiency fractures, of whom 40 (40%) had bilateral symmetric lesions of the sacral alae and eighty-five patients (85%) had sacral involvement, Kwon et al. (**E**) The distribution of PIF involvement was as follows, according to Abe et al.: sacroiliac joint in 61% (sacral ala in 53% and a medial region of ilium in 8%), upper sacrum (S1 -S2) in 28%, lower sacrum (S3-S5) in 4%, pubis in 4%, and ischium in 3%. The non-irradiated iliac wing was never affected. 23 (85%) of the 27 patients had more than one area of increased activity

According to the findings of several studies, the distribution of pelvic insufficiency-related fractures is shown in Fig. 3. Table 1 provides a review of relevant research on probable fracture locations, including details about each finding.

**Table 1:** Overview of literary works on possible fracture locations

Ref	Ration of PIF Patients	RT Dose (Gy)	Percentage of incidence (years cumulative incidence)	Number of symptomatic patients	Fracture sites									Multiple fractures
					lumbar vertebrae	Sacrum body	sacroiliac joint	Sacrum and pubis	iliac crest	sacrum and acetabulum	Acetabulum	Pelvic bone	Femoral neck/head	
[37]	08/463	50.4–55.8	1.7	08	02	NR	08	NR	NR	NR	NR	NR	NR	02
[38]	57/335	45–50	17	47	NR	40	48	NR	NR	NR	02	09	NR	NS
[6]	18/158	50	11.4	NR	4	05	13	NR	NR	NR	1	11		12
[39]	29/300	45	9.7	13	NR	NR	24	3	1	1	NR	NR	NR	04
[22]	100/510	30.6–66.6	45.2 (5)	43	NS [40% had bilateral symmetric lesions of the sacral alae. 85% had sacral involvement]									61
[3]	16/235	55	9.5 (5)	11	NR	NR	12	NR	03	NR	NR	03	01	02
[40]	33/99	50.4	34	20	14	15	04	NR	01	NR	NR	14	02	NS
[36]	21/59	50	36.9	09	01		32	NR	NR	NR	02	09	NR	16
[35]	11/78	50.4	NS	07	NR	NR	15	NR	NR	NR	NR	04	NR	05
[19]	20/101	45–50	20	10	NS									11
[41]	11/61	45-50.4	18	03	NS									NS
[32, 42]	84/533	50–50.4	15.8	∼ 68	25	39	22		11	NR	NR	27	04	51
[20]	05/183	45–50	2.7	05	NS									NS
[34]	83/557	30.6–54	19.7 (5)	48	NR	27	79	NR	NR	NR	03	08	NR	51

Ref = Reference, NR = not reported, NS = not specified

### Justification for awareness or avoidance of pain in PIF following RT

PIFs involving the pelvis are often overlooked, although they are becoming increasingly understood as a key contributor to lower back, buttock, and groin discomfort in elderly women. Patients with PIF report pelvic pain after pelvic radiotherapy for cervical cancer, which is related to radiological abnormalities such as hot uptakes on bone scans or pelvic bone fractures by plain radiography or CT scan. Numerous investigations provide credence to the assertion that the first symptom experienced by each patient was discomfort in the pelvic region and that this pain was a persistent complaint on the part of the patients that lasted for several months (Table 2). According to Schmeler et al., most fractures (83%) were discovered within two years following the end of treatment [39]. Although most reports of discomfort focus on the lower back or hips, there is some proof that pain can be radiated down the legs [38].

Even though PIF can induce symptoms such as pelvic pain, there may be minimal or no symptoms in some people. In a study carried out by Ogino et al. out of 335 patients, 57 acquired PIF. Of these 57 patients, 47 presented low back pain, while 10 were asymptomatic [38]. According to Tokumaru et al. and Kwon et al., 23–57% of participants with PIF do not exhibit any indications [22, 36]. Blomlie et al. reasoned that since all patients without pain had smaller lesions (< 1 cm^2^) on MRI and it was postulated that minor fractures might not be painful, the size of the lesions would be related to the intensity of the symptoms [33]. According to another study, patients with symptoms were more likely than those without symptoms to develop PIF in various places along their pelvic bone [36].

Taken together, radiation induced PIF is a common side effect of standard radiation therapy for uterine cervical cancer. After complete pelvic radiation therapy for gynecological malignancies, if patients report pelvic pain, we must always take into account PIF. In addition to that, a time-dependent follow-up examination is also strongly recommended due to the predominance of asymptomatic individuals. When PIF is found and treated early, the quality of life of people with cervical cancer can be improved and unnecessary medical costs can be cut. However, not all patients who experience pain after radiation therapy end up with PIF. This was demonstrated in a study by Ikushima et al., in which 33 of 158 patients reported pelvic pain during the follow-up period after RT, but only 18 of these patients went on to develop PIF [6]. Due to this reason, it is impossible to say with absolute certainty that PIF will only occur in the presence of pain or that PIF will not occur in the absence of pain.

**Table 2:** Onset and timeframe of discomfort in patients with cervical cancer following radiotherapy

Authors	Average onset of symptoms	Duration of symptoms	Reference
(Ishikawa et al., 2021)	05–51 months	03–20 months	[43]
(Ioffe et al., 2014)	29 months	NR	[44]
(Park et al., 2011)	12.5 months	∼ 07 months	[3]
(Schmeler et al., 2010)	14.1 months	NR	[39]
(Kwon et al., 2008)	16.9 months	1 to 32 months	[22]
(Oh et al., 2008)	13 months	NR	[34]
(Ogino et al., 2003)	NR	0–35 months	[38]
(Huh et al., 2002)	12 months	01–11 months	[37]
(Moreno et al., 1999)	13.7 months	1 to 13 months	[5]

NR = not reported, (∼) = approximately

### Is PIF exclusively seen in patients with cervical cancer due to RT?

PIFs are characterized by bone failure under physiological loads, as suggested by the definition of the term. Therefore, it follows that anything that decreases bone mass could be a contributor. Without a doubt, osteoporosis is the most common etiology. Menopause, being an older woman, taking glucocorticoids, using heparin, a history of smoking, mechanical changes after hip arthroplasty, secondary hyperparathyroidism, hypocalcemia, and other conditions have all been linked to PIF [9, 22, 26, 43, 45, 46].

The presence of concurrent radiation therapy, rheumatoid arthritis, osteoarthritis, renal failure, hormone replacement therapy (HRT) or any combination of these conditions in several of the women further increased their bone susceptibility to fracture [20]. When treating patients with cervical cancer, radiation therapy is often used, either as the sole treatment or as postoperative irradiation. According to the findings of Sakaguchi et al., women who received radiation therapy had a higher risk of pelvic fracture compared to women who did not receive radiation therapy [41]. In one study, Baxter et al. found that pelvic radiation tripled the incidence of pelvic fractures in female anal cancer patients (HR = 3.16) [13]. As a direct result of this, pelvic radiation, which is used to treat various forms of cancer, is also a risk factor for the eventual development of insufficiency fractures.

Disease survival has improved due to the introduction of cisplatin chemotherapy and developments in RT, increasing the importance of therapeutic issues. Data on the long-term side effects of radiation therapy, specifically the impact of intensity-modulated radiation therapy (IMRT) on the pelvic girdle of women with cervical cancer, are scarce. Ioffe et al. conducted a study in 2014 that showed that IMRT is less likely to cause pelvic girdle than traditional RT [44]. Although Shih et al. observed that PIF rates were 4.9% regardless of whether patients received IMRT or conventional RT, they stated that the use of IMRT did not reduce PIF [35].

Several investigations have also revealed that, in this case, concomitant chemotherapy did not have a significant impact on the emergence of PIF [3, 34]. Likewise, neither radiation exposure nor surgery was significantly linked to the likelihood of PIF [34]. Mehmood et al. found that, although treatment was not linked to fracture formation, cervical cancer patients experienced much more insufficiency fractures and bone pain than uterine cancer patients. This raises the possibility that concomitant chemotherapy may have a significant role in these individuals’ increased risk of insufficiency fractures and bone morbidity in these individuals and emphasizes the need for more research to find, stop and reduce these long-term side effects [47].

No correlation was observed between PIF and receiving more than four cycles of chemotherapy in the study by Ramlov et al. [19]. In a study comparing patients treated for locally advanced cervical cancer before and after the introduction of concurrent chemotherapy, Gondi et al. examined all serious late toxicities after radiation therapy or chemoradiation therapy [48]. They discovered a strong correlation between PIF and chemotherapy treatment. Despite numerous studies that examined the link between chemotherapy and PIF, no conclusive evidence of a causal relationship has yet been found [3, 35, 39, 40, 49].

Of course, the objective of radiation therapy is to destroy the tumor with the least amount of damage to the surrounding healthy tissue. Although PIF has been described as a rare complication in the era of megavoltage RT equipment, the true incidence of PIF following RT is unknown. That is why, considering all published clinical results, it is essential to keep in mind that radiation is not the sole culprit behind every case of pelvic insufficiency fracture. In a research carried out by Cabarrus et al., only 29 (20%) of the total of 145 patients who had radiological symptoms of PIF had previously undergone pelvic RT [50]. However, a recent study with a hazard ratio of 1.65 to 3.16 demonstrated that RT significantly increases the risk of fracture [13]. Furthermore, several studies indicated that the cumulative incidence of PIF following RT in cervical cancer ranged from 8.2 to 45.2% [6, 13, 22, 34, 38].

For a deeper dive into the meaning of this empirical evidence, researchers argue that patient attributes (such as gender, menopausal status, age, body weight, and comorbidities) and treatment parameters (such as RT volume, dose per fraction, total dose, RT technique, and chemotherapy use) influence the progression of PIF. Therefore, it is impossible to isolate the impact of RT on bone injury without considering the confounding factors that increase the risk of PIF.

### Potentially influencing factors in the development of PIF upon radiotherapy

After conducting a review of relevant research, it has become abundantly clear that factors such as menopausal state, weight, BMI, age, and various treatment and diagnosis approaches are potential risk factors for osteoporosis that are closely associated with the development of PIF in patients who have undergone RT for cervical cancer.

#### Menopausal status

Postmenopausal women, according to many studies, are at a higher risk for PIF than their younger counterparts [21, 37, 39]. Kim et al. (2012) found that osteoporosis and female sex were independent risk factors for sacral insufficiency fractures (SIF) after chemoradiation, emphasizing the close link between osteoporosis risk factors and the development of insufficiency fractures [51]. Ramlov et al. found that the incidence of PIF was 6% (3 out of 52) and 35% (17 out of 49) in premenopausal and postmenopausal patients, respectively, with a strong correlation between hormone replacement treatment and PIF in premenopausal individuals [19]. The results of a multivariate analysis conducted by Yamamoto et al. indicated that postmenopausal status, the presence of rheumatoid arthritis, and the use of high-dose intracavitary brachytherapy (HDR-ICBT) were all significant risk factors for the development of PIF [32].

#### Body weight and BMI

Studying postmenopausal patients with intact uterine cervical cancer treated with radiation therapy, Ogino et al. found that a body weight of 49 kg or less and more than three deliveries were identified as having a significant effect on the insufficiency fracture [38]. Oh et al. found weight less than 55 kg to be a major risk factor for pelvic insufficiency fractures in Korean women receiving pelvic radiation [34]. In a study of women with cervical cancer treated with curative intention radiation (no patients had pre-existing pelvic fractures, pelvic fractures identified on pretreatment imaging, or bony metastases), Schmeler et al. found that pelvic fractures were related to a lower BMI [39]. What additional effects radiation has on low BMI female patients is still unknown; however, body mass index (BMI) is another factor that should be considered when determining the risk of PIF. This is probably because these people have lower levels of both body fat and free circulating estrogen, both of which play a role in preventing bone loss [39]. However, the potential side effects of RT on underweight women are still debatable. With an average BMI of 23 and 22, respectively, for the general population, Korea and Japan had the highest prevalence of PIF in gynecologic malignancies [22, 36, 52, 53]. In the Shih et al. trial, patients who developed PIF had lower mean BMI (25.9; range, 17.8–34.4) than patients who did not (27.2; range, 18.2–57.9) [35]. Similarly to this, in a research by the MD Anderson Cancer Center, the median BMI was lower in people who developed PIF compared to those who did not (median BMI, 27.6; range, 15.5–58.2) in 300 patients with cervical cancer (26 vs. 28) [39]. However, research by Ramlov et al. found no correlation between having a low BMI and being at risk for health problems [19].

#### Age

With the aid of univariate analysis, Uezono et al. identified significant risk factors as being older than 70 years and having a lower bone marrow CT value [40]. According to the same analysis, Park et al. discovered that women over 75 years of age, with a BMI under 23, a bone mineral density (BMD) score below − 3.5 SD, and who had their first positron emission tomography/computed tomography (PET/CT) within a year after finishing radiotherapy had a significantly higher risk of developing PIF [3]. But when Sakaguchi et al. performed a multivariate analysis, they discovered that an abnormal body mass index (BMI) (more than 25 kg/m^2^ or less than 18 kg/m^2^) and the administration of five or more chemotherapy cycles were independently related to SIF [41].

Based on multivariate analysis with the Cox risk test, Tokumaru et al. concluded that patients with cervical cancer with greater age (> 70 years) and low body weight (< 50 kg) may be at risk for the development of pelvic PIF after pelvic radiation therapy [36].

#### Other factors

The weighted dose of relative biological effectiveness (RBE) (DRBE 50%), according to another study by Mori et al., was associated with an increased risk of SIF. When age > 50 years was taken into account, only current smoking behavior contributed to SIF; neither linear energy transfer dose (LETd) nor physical dose characteristics were significant risk factors [54]. The study by Ramlov et al. found that PIFs are related to the dose and volume of irradiation to the elective pelvic target, rather than with lymph node boosts. In some cases, the risk of PIF might be drastically reduced by decreasing the prescribed elective dose from 50 to 45 Gy [19].

Low bone mineral density (BMD) appears to be one of the predisposing variables that can lead to the development of PIF following radiation [55]. In particular, they found that sacral bone BMD was lower in the PIF group, at 127.8 mg/cm^3^, compared to the other group’s BMD of 173.1 mg/cm^3^. The mean BMD of the lumbar vertebrae was lower in the PIF group (87.9 mg/cm^3^) compared to the other group (121.4 mg/cm^3^). In their analyzed cases, the dose did not appear to play a significant role in the occurrence of PIF. However, Oh et al. proposed that a radiation dose of 50.4 Gy could act as a predisposing factor [34].

The widespread use of imaging modalities during follow-up, such as CT, MRI, and bone scintigraphy, may accelerate the detection of asymptomatic PIF. The frequency of PIF after RT is further affected by imaging examinations to detect the condition. Research using magnetic resonance imaging found that 89% of patients had PIF-consistent findings that were consistent with PIF after RT [33], but a study using bone scintigraphy found that 34% of patients had such findings [21].

Damage to the microvasculature of mature bone is one of the primary causes of the late effects on radioactive bone. This leads to microcirculation blockage, which damages the periostic vasculature and osteoblastic function. As a result, there is an increased risk of suffering traumatic or stress fractures; however, because bone has a slow growth rate, the effects of injury effects take time to manifest [5].

### Variables not affecting post-RT PIF growth

According to Schmeler et al., there were no statistically significant differences were found when examining factors such as ethnicity, smoking history, histology, stage of cancer, tumor grade, radiation type, radiation dose, or usage of concomitant treatment [39]. However, in a multivariate and univariate examination of factors associated with symptomatic PIF, Ogino et al. reported that age, type II diabetes, menopause age, external dosage, and total brachytherapy did not contribute substantially [38]. Cooper et al. further noted that unlike many metastatic diseases, PIF has not associated with the development of soft tissue growth or osteolytic lesions [31]. There is an exception to this rule, however, and that is parasymphyseal fractures, which can be accompanied by the formation of soft tissue, which can sometimes have a pseudo-malignant look. Additional research is necessary to evaluate whether these factors make a substantial difference in the progression of PIF in patients with cervical cancer who have undergone radiation.

### Preventive measures to minimize the severity of PIF

Oh et al. suggested two strategies to reduce PIF’s likelihood [34]. First, osteoporosis treatment can mitigate radiation’s deleterious effects; second, bone strengthening can fortify the skeletons themselves. Bisphosphonate is an effective drug for treating osteoporosis by Sambrook et al. [56], and it has also been shown to be useful in reducing cancer-induced bone loss by Guise et al. [57].

In the case of patients being treated for cervical cancer by hysterectomy and double anexectomy, many individuals develop estrogen-deficiency-dependent osteoporosis before beginning pelvic irradiation. There are a variety of female-specific variables that might lead to osteoporosis after menopause. The use of corticosteroids and heparin both increases the risk of fractures in people with osteoporosis. Too much thyroid hormone production has also been associated with decreased bone mineral density in postmenopausal women. Most osteoporosis medications work by reducing bone resorption, including estrogens, biphosphonates, and calcitonin. The primary line of treatment for osteoporosis is estrogen replacement. It reduces the risk of fractures, and this positive effect is especially pronounced in women who started hormone replacement treatment within five years of menopause [58]. Combining the effects of estrogen agonists and antagonists, raloxifene is a novel therapy for osteoporosis. Both bone mineral content and bone resorption have been shown to increase with raloxifene treatment. On top of that, it does not promote endometrial expansion [59]. However, this is not the same as fracture mitigation, as happens with tamoxifen and fluoride [60]; so, fracture studies are necessary to support this claim.

Painful PIFs can be treated with cementoplasty, which is widely recognized as an effective treatment for osteoporotic insufficiency fractures, as well as metastatic spine and pelvic disease [61], particularly sacroplasty, a relatively recent therapeutic option for fractures of the sacrum that are caused by insufficiency [62]. This procedure, which can quickly and efficiently relieve pain, is also an option for treating other conditions. Despite the lack of data on, especially radiation-related insufficiency fractures, this treatment has been shown to be successful [63, 64].

A large percentage of bone defects caused by PIF do not require surgery. There is, however, the possibility of undergoing surgery in order to address PIF [35]. Treatment often involves pain management with medication and physical therapy, as well as the use of assistive devices (such as a walker) to alleviate pressure on the pelvic region.

The majority of symptomatic patients were able to achieve full resolution after receiving conservative treatment, which included the use of analgesics (painkillers) and rest. Studies have shown that conservative therapy, such as rest and nonsteroidal anti-inflammatory drugs (NSAIDs), can help alleviate pelvic pain, as evidenced in a study by Kwon et al., where 43 patients (43%) experienced pelvic pain at the time of MRI [22]. All patients experienced symptom relief, which lasted from about one month to three years. Those who received follow-up MR scans had their fracture lines mend and the hypointense reactive bone marrow alteration subside. As time passed, the fracture line faded and became undetectable.

However, certain patients require narcotics or hospitalization due to severe pain and disability. These patients typically have multiple fracture sites or larger lesions [33, 34]. There is some evidence that pentoxifylline can help patients recover from their symptoms [65]. However, there is little evidence that any one drug can effectively treat PIF. Some pharmaceutical treatments, such as medroxyprogesterone acetate, vitamin D-rich calcium supplements, and bisphosphonates, have been shown to speed up the healing process of fractures, as reported by Tai et al. (2000) [66]. More research is needed to see if it can help minimize the chance of PIF in people who already have risk factors, including advanced age and underweight. A summary of the findings from the review of the published literature is given in Table 3, along with the number of individuals who were admitted to the hospital and the management employed to treat the severity of PIF.

**Table 3:** List of medications used to manage the severity of PIF and the percentage of hospitalized patients

Study	PIF patients number	Percentage of hospitalized patients (Patient number)	Medications (Patient number)	Ref
(Ishikawa et al., 2021)	18	44.45 (08)	Analgesics (13), bed rest and non-steroidal anti-inflammatory drugs	[43]
(Sakaguchi et al., 2019)	61	00	Non-steroidal anti-inflammatory drugs and rest	[41]
(Yamamoto et al., 2017)	84	7.14 (06)	NSAIDs (25), an opioid combined with NSAIDs (20)	[32]
(Shih et al., 2013)	78	00	Observation (6), bisphosphonate (4), and surgery (1)	[35]
(Tokumaru et al., 2012)	21	00	Rest or non-narcotic analgesic drugs	[36]
(Uezono et al., 2011)	22	00	Opioids	[40]
(Park et al., 2011)	16	6.25 (01)	Not specified	[3]
(Kwon et al., 2008)	100	00	Nonsteroidal anti-inflammatory drugs	[22]
(Oh et al., 2008)	83	13.3 (11)	Narcotic medications, rest and nonsteroidal anti-inflammatory drugs	[34]
(Ogino et al., 2003)	57	10.81 (08)	Non-narcotic analgesics (23), narcotic analgesics (05)	[38]
(Moreno et al., 1999)	08	00	Rest and non-steroidal anti- inflammatory drugs	[5]

Formula to calculate the percentage of hospitalized patients:

$${\rm{Percentage}}\,{\rm{of}}\,{\rm{hospitalized}}\,{\rm{patients}} = \frac{{{\rm{Number}}\,{\rm{of}}\,{\rm{hospitalized}}\,{\rm{patients}}}}{{{\rm{Number}}\,{\rm{of}}\,{\rm{PIF}}\,{\rm{patients}}}} \times 100$$

## Limitations and recommendations

Our comparative review of the literature on PIF after radiation for cervical cancer patients revealed substantial heterogeneity in terms of study outcomes, including prevalence rate, associated risk factors, symptoms, severity, diagnosis, preventive interventions and follow-up periods. Despite the importance of their investigation, there were several caveats. Most studies were conducted using a retrospective design, and radiologists were unable to predict the stage of each fracture because some patients were discovered only by routine follow-up imaging exams. Due to the nature of this investigation, the researchers were unable to obtain identical imaging studies for all patients at the same time. In addition, there was a lack of completeness in the toxicity data for some of the patients, which was poorly reported when it was present or absent altogether. The fact that not all patients had access to accurate information on their use of hormone replacement therapy and other drugs that can affect bone mineral density may have led to an underestimation of the true risk of fracture among those receiving pelvic radiotherapy. Consequently, women who receive pelvic radiation for cervical cancer should consider bone mineral density testing and pharmacological intervention.

Another constraint of our research is the restricted examination of the possible role of early menopause after surgery or radiation therapy for cervical cancer in women who are not yet menopausal. Although this is a crucial factor to take into account when analyzing the larger effects of cervical cancer treatment, the lack of information made it difficult for us to fully address this subject within the parameters of our study.

On the other hand, referral bias may exist because the data for some research came from a single peripheral area or dealt with a very small number of patients. The incidence of fractures or the increase in bone density during the trial may have been affected by the fact that many physicians treated all patients as soon as they identified vitamin D deficiency, osteopenia, or osteoporosis. The short follow-up periods in certain studies have also made it difficult to report long-term morbidity related to osteoporosis and PIF. However, several studies neglected to assess patients’ quality of life or other reported outcomes. It is common knowledge that cancer patients who make it through treatment often face serious challenges due to the toxicity and lasting effects of their care. As a consequence of this, it is strongly recommended that additional research be conducted on bone toxicity to determine the impact on cervical cancer survivorship and to determine to what extent the findings can be generalized to a broader population.

This study has several strengths that contribute to its robustness and reliability. First, the comprehensive review of the literature conducted allowed for a thorough examination of existing research on PIF after radiation therapy for cervical cancer. Using multiple online resources and employing a systematic search strategy, a wide range of relevant articles were identified and included in the review, improving the comprehensiveness of the study. Additionally, inclusion criteria were carefully defined to ensure selection of studies with high relevance to the topic, thereby minimizing the risk of bias and ensuring the reliability of the findings. Furthermore, the synthesis of findings from various studies provided a comprehensive overview of the incidence, risk factors, clinical manifestations, and management strategies of PIF, allowing a deeper understanding of this important clinical issue.

## Conclusion

In fact, PIFs are a significant concern after radiation therapy for cervical cancer. This study provides a comprehensive overview of the literature on PIF following cervical cancer radiotherapy, synthesizing evidence from various studies to elucidate the underlying mechanisms, risk factors, and management strategies. Based on a comprehensive review of multiple studies, it is evident that PIF is a common complication, with cumulative incidence rates ranging from 8.2 to 45.2%. The sacrum emerges as the most frequently implicated site for fractures, with up to 77% of cases involving sacral fractures. Furthermore, research indicates a robust correlation between PIF and variables such as age, body weight, BMI, and menopausal state. It should be noted that PIF incidence rates might approach 35% in postmenopausal women, who are at increased risk of developing the condition. Having said that, the review underscores the multifactorial nature of PIF development, involving not only radiation therapy, but also osteoporosis and other comorbidities. Despite these challenges, early detection and preventive measures, including osteoporosis treatment and bone strengthening, offer promising avenues to mitigate the severity of IPF and improve patient outcomes. These findings underscore the importance of proactive management strategies and highlight the need for continued research to address the complexities surrounding PIF in cervical cancer survivors.

## Data Availability

All data generated or analyzed in the study are involved in this published article itself.

## Abbreviations

BMD: Bone mineral density
CT: Computerized tomography
HRT: Hormone replacement therapy
IMRT: Intensity-modulated radiation therapy
LETd: Linear energy transfer dose
MRI: Magnetic resonance imaging
NSAIDs: Non-steroidal anti-inflammatory drugs
PET: Positron emission tomography
PIF: Pelvic Insufficiency Fracture
RBE: Relative biological effectiveness
RT: Radiation therapy
SIF: Sacral Insufficiency Fractures
